# Free Radical Scavenging, Anti-inflammatory and Antibacterial Activity of Acorus calamus Leaves Extract Against Pseudomonas aeruginosa and Staphylococcus aureus

**DOI:** 10.7759/cureus.55987

**Published:** 2024-03-11

**Authors:** Pranav Haran, Rajeshkumar Shanmugam, Pavithra Deenadayalan

**Affiliations:** 1 Nanobiomedicine Lab, Center for Global Health Research, Saveetha Medical College and Hospital, Saveetha Institute of Medical and Technical Sciences, Chennai, IND

**Keywords:** antimicrobial properties, membrane stabilization, anti-inflammatory properties, antioxidant properties, acorus calamus dmso extract

## Abstract

Background

Herbal medicine, or phytotherapy, has been used for centuries in traditional healing practices to harness the therapeutic properties of different plant-derived elements. *Acorus calamus*, a perennial herbaceous plant, has significant historical importance in traditional medicine, specifically in Ayurveda, where it is referred to as “Vacha.” This study investigates the antioxidant, anti-inflammatory, and antimicrobial characteristics of the *A. calamus* dimethyl sulfoxide (DMSO) extract. The objectives of the research are to provide valuable knowledge about the preparation of *A. calamus* DMSO extract and to explore its potential anti-inflammatory, antioxidant, and antimicrobial effects.

Materials and methods

The *A. calamus* DMSO extract was derived from leaves, and its antioxidant activity was evaluated through the use of the 2,2-diphenyl-1-picryl hydrazyl (DPPH) assay, hydroxyl radical scavenging assay (H_2_O_2_ assay), and ferric reducing antioxidant power (FRAP) assay. The anti-inflammatory activity was assessed using the Bovine serum albumin (BSA) denaturation assay, egg albumin (EA) denaturation assay, and membrane stabilization assays. The antimicrobial activity was analyzed using the agar well diffusion technique and the time-kill curve assay.

Results

In DPPH and H_2_O_2_ tests, the DMSO extract of *A. calamus *showed significant antioxidant activity, near that of standard ascorbic acid. The FRAP assay demonstrated a correlation between the dose and the activity of reducing ferric ions. The *A. calamus *DMSO extract exhibited significant anti-inflammatory properties in BSA and EA denaturation assays, similar to the standard diclofenac sodium. The anti-inflammatory potential of the *A. calamus* DMSO extract was further confirmed through the membrane stabilization assay. The DMSO extract of *A. calamus* exhibited a significant inhibition zone against the pathogens *Streptococcus mutans *and *Pseudomonas aeruginosa* during the antimicrobial evaluation, surpassing the efficacy of the standard antibiotic. The time-kill curve assay validated the antibacterial efficacy, which was dependent on the concentration.

Conclusion

The *A. calamus *DMSO extract exhibited promising antioxidant, anti-inflammatory, and antimicrobial properties, supporting its traditional use in alternative medicine. The findings suggest its potential as a natural resource of compounds with bioactive properties for use in pharmaceutical and nutraceutical applications.

## Introduction

Herbal medicine, also called phytotherapy, includes an extensive array of plant derivative elements like roots, leaves, seeds, and flowers and individually exhibits bioactive compounds [1]. Plant-based remedies are used as herbal medicine for healing practices like treating, preventing, and alleviating various medical conditions [2]. The utilization of plants for therapeutic purposes predates documented history, with convincing proof indicating their extensive usage across different eras and civilizations. Herbal medicine comprises a diverse range of substances derived from plants, such as leaves, roots, seeds, and flowers, each containing distinct bioactive compounds [3]. Phytocompounds derived from plants are recognized for their multifaceted properties, including antioxidative, antibacterial, anti-inflammatory, and antipyretic effects [4].

*Acorus calamus*, often referred to as sweet flag or calamus, is a perennial herbaceous plant that is well known for its historical and current importance in traditional medicine and various cultural customs [5]. This plant, belonging to the *Acoraceae* *family*, can be recognized by its tall, sword-shaped leaves and a uniquely fragrant rhizome. The use of *A. calamus* has been prevalent in numerous cultures for centuries, and its therapeutic properties have been extensively recorded in scientific literature [6]. In the ancient Indian medical system of Ayurveda, *A. calamus* is referred to as “Vacha” and is practically used for its potential cognitive and neuroprotective benefits. Substantial research has investigated the pharmacological properties of this substance, particularly its capacity to augment memory and cognitive functions [7]. *A. calamus* exhibits excellent antioxidant properties, which are essential for counteracting harmful free radicals inside the body. The plant's ability to neutralize harmful free radicals has been prominently featured in numerous scientific studies, highlighting its potential to mitigate oxidative stress [8]. Studies also explored the anti-inflammatory effects of *A. calamus*, suggesting its potential for alleviating inflammatory conditions. The phytochemical composition of the plant, including compounds like β-asarone, plays a role in its anti-inflammatory properties [9]. It has also been recognized for its antimicrobial activity against various pathogens. The plant's bioactive compounds, such as β-asarone, contribute to its antimicrobial efficacy. The antimicrobial potential of *A. calamu*s extracts provides insights into their efficacy against microbial strains [10]. Nevertheless, there have been minimal studies based on the dimethyl sulfoxide (DMSO) extract of *A. calamus*. Thus, this research study focused on the potential of the DMSO plant extract of *A. calamus* in terms of antioxidant, anti-inflammatory, and antimicrobial properties.

By using the *A. calamus *DMSO extract for its inherent antioxidant, anti-inflammatory, and antimicrobial properties, this study aims to unravel the potentiality of the *A. calamus* DMSO extract, which can be harnessed for potential applications in health and related fields. The insights gained from evaluating and comparing the antioxidant, anti-inflammatory, and antimicrobial activities of medicinal plants contribute to our understanding of their natural sources. This study aims to explore the inherent antioxidant, anti-inflammatory, and antimicrobial properties of the *A. calamus* DMSO extract, aiming to unveil its potential applications in health and related fields. The investigation into and comparison of the antioxidant, anti-inflammatory, and antimicrobial activities of medicinal plants contribute valuable insights to our comprehension of natural sources. These findings present a piece of promising evidence for the development of innovative alternatives in antioxidants, anti-inflammatories, and antimicrobials, with broader implications for sustainable and eco-friendly therapeutic applications [11].

The primary objective of this research is to prepare the *A. calamus* DMSO extract, assess and compare its antioxidant activity using the 2,2-diphenyl-1-picryl hydrazyl (DPPH) assay, the hydroxyl radical scavenging assay (H_2_O_2_), and the ferric-reducing antioxidant power (FRAP) assay. Anti-inflammatory activity was evaluated through bovine serum albumin (BSA) denaturation, egg albumin (EA) denaturation, and membrane stabilization assays, while antimicrobial activity was determined using agar-well diffusion techniques and time-kill curve assays. The insights derived from these evaluations will hold potential implications for applications in health and related fields, highlighting the role of harnessing the therapeutic properties of medicinal plants [12].

## Materials and methods

Preparation of plant extract

To prepare the plant extract, 10 grams of *A. calamus *leaves were meticulously weighed and washed with water to eliminate dust particles. Subsequently, the leaves were crushed, and 25 ml of DMSO extract was added. The resulting mixture was then subjected to an ultra-digital sonicator for 30 minutes. Following the sonication process, the leaves were transferred to an orbital shaker and agitated for an additional 30 minutes. The obtained extract was then filtered, and the filtrate was reserved for further research purposes.

Antioxidant activity

DPPH Assay

The DPPH assay was performed following the methodology detailed in a prior study [13]. Our examination focused on assessing the antioxidant activity of the *A. calamus *DMSO extract, utilizing the DPPH assay for this specific evaluation.

H_2_O_2_ Assay

The study followed the methodology laid out in prior research conducted by Shanmugam et al. to evaluate the efficacy of the *A. calamus* DMSO extract as an antioxidant, utilizing an H_2_O_2_ assay [14].

FRAP​​​​​ Assay

This assay was demonstrated using previous research conducted by Viswanathan et al. to analyze the *A. calamus *DMSO extract antioxidant activity using the FRAP assay [15].

Anti-inflammatory

BSA Denaturation Assay

The anti-inflammatory activity of *A. calamus *DMSO extract was evaluated using the modified method of Ameena et al. [16]. BSA is utilized in this assay to assess the denaturation potential of *A. calamus* DMSO extract.

EA Denaturation Assay

Utilizing a study by Ameena et al. method, the assessment of the anti-inflammatory activity of the *A. calamus* DMSO extract involved a series of steps [16]. The assay was incorporated using 0.2 mL of fresh EA, 2.8 mL of phosphate-buffered saline (PBS) at pH 6.4, and 0.6 mL of the *A. calamus* DMSO extract at varying concentrations dissolved in 0.2% DMSO. The EA in this assay was used to examine the denaturation efficacy of* A. calamus* DMSO extract.

Membrane Stabilization Assay

The in vitro membrane stabilization assay is a commonly utilized technique to evaluate the membrane-stabilizing properties of medicines. This experiment evaluates a drug's capacity to stabilize the cell membrane, therefore inhibiting its disruption and the resultant release of intracellular contents. Required materials: human red blood cells (RBCs), Tris-hydrochloride (Tris-HCl) buffer (50 mM at pH 7.4), and PBS. Multiple concentrations of *A. calamus *DMSO extract (ranging from 10 to 50 µg/mL) were created, with saline solution and distilled water used as controls.

Anticoagulant-containing fresh human blood was obtained in a sterile tube. The blood was centrifuged at room temperature for 10 minutes at 1,000 g to separate red blood cells from other components. The liquid above the sediment was removed, and the red blood cells were washed three times with phosphate-buffered saline (PBS). The RBCs were then suspended in Tris-HCl buffer to create a 10% (v/v) RBC suspension. 1 mL of the RBC suspension was pipetted into each centrifuge tube, followed by the addition of different doses of A. calamus DMSO extract. The mixture was gently mixed and incubated for 30 minutes at 37°C. The centrifuge tubes were spun at 1000 g for 10 minutes at room temperature to collect the red blood cells. The supernatant's absorbance was measured at 540 nm with a UV spectrophotometer. The percentage inhibition of hemolysis was calculated.

Antimicrobial activity

Agar Well Diffusion Method

The antimicrobial activity of *A. calamus* DMSO extract was evaluated using the agar well diffusion technique. Mueller Hinton agar plates were prepared and sterilized using an autoclave at 121^o^C for 15-20 minutes. After sterilization, the medium was poured onto the surface of sterile Petri plates and allowed to cool to room temperature. The bacterial suspension (*P. aeruginosa *and *S. aureus*) was spread evenly onto the agar plates using sterile cotton swabs. Wells of 9mm diameter were created in the agar plates using a sterile polystyrene tip. The wells were then filled with different concentrations (25 µg, 50 µg, 100 µg) (12). For Bacteria, Amoxyrite was used as a standard. The plates were incubated at 37°C for 24 hours. The antimicrobial activity was evaluated by measuring the diameter of the inhibition zone surrounding the wells. The diameter of the zone of inhibition was measured using a ruler and recorded in millimeters (mm) and the zone of inhibition was calculated. The measurement of the zone of inhibition was carried out using a ruler, and the results were recorded in millimeters. This measurement served as an indicator of the antibacterial efficacy of the DMSO plant extract derived from *A. calamus*.

Time-Kill Curve Assay

A 1 mL aliquot of the bacterial suspension (*P. aeruginosa* and *S. aureus*) was added to 9 mL of Mueller Hinton broth containing the *A. calamus* DMSO extract at a concentration of 25 µg, 50 µg, and 100 µg. The final microbial concentration was approximately 106 CFU/mL. The mixture was then incubated at 37°C with shaking at 200 rpm for varied time intervals (0, 4, 6, 8, 10, 12, and 24hr). Then the percentage of dead cells is calculated at wavelength of 600nm at regular time intervals.

Statistical analysis

All the experiments were performed in triplicates. The error bars provided in the graphical representation are represented as means ± SEM (Standard Error of Mean) analysis. Welch's correction test was performed using the software GraphPad Prism. The significance level (p-value) was expressed as <0.05.

## Results

Antioxidant activity

DPPH Assay

**Figure 1:**
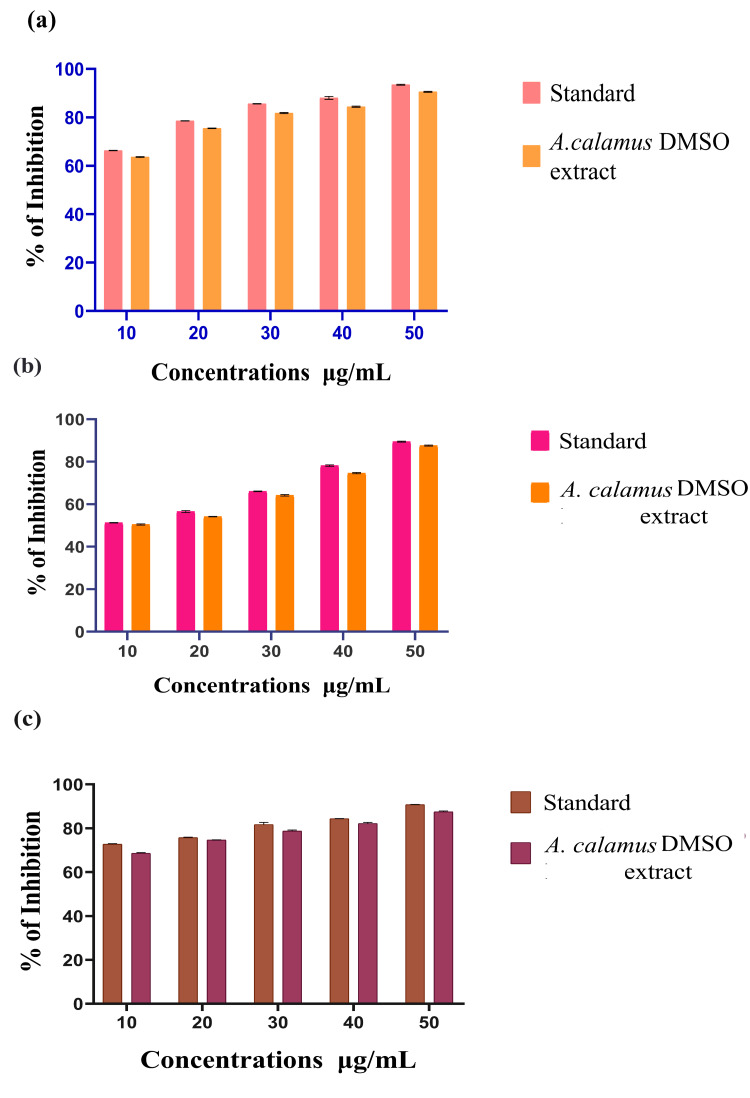
Antioxidant activity of A. calamus DMSO extract (a) DPPH assay (b) H2O2 assay, and (c) FRAP assay A. calamus: *Acorus calamus*; DMSO: Dimethyl sulfoxide; DPPH: 2,2-diphenyl-1-picrylhydrazyl; H_2_O_2_: Hydrogen peroxide; FRAP: Ferric-reducing antioxidant power The values are provided in triplicates and the error bars present in a graphical representation of DPPH, H_2_O_2_, and FRAP assays indicate the standard error of mean (SEM). The statistical test used was Welch's correction. A value of P≤0.05 was considered to be statistically significant as compared to the standard group.

In Figure 1a, the graph depicts the antioxidant activity of the *A. calamus *DMSOextract and a standard (ascorbic acid) against DPPH radicals. At the highest concentration of 50 µg/mL (microgram per milliliter), the standard displays a 93.15% inhibition in radical activity, whereas the *A. calamus* DMSO extract shows a slightly lower 90.48% inhibition. In comparison, at the lowest concentration of 10 µg/mL, the standard exhibits a 66.25% inhibition, and the DMSO extract of *A. calamus* shows a slightly lower effect of 63.94% inhibition.

H_2_O_2_ Assay

In Figure 1b, the graph illustrates the *A. calamus *DMSO extract and standard ascorbic acid antioxidant activity using an H_2_O_2 _assay against H_2_O_2 _radicals. At a maximum concentration of 50 µg/mL, the standard exhibits an inhibition rate of 89.9%, whereas the *A. calamus* DMSO extract shows a somewhat lower inhibition rate of 87.4%. In the concentration of 10 µg/mL, the standard percentage was 51.2%. Whereas DMSO *Acorus calamus *extract shows 50.3%, which is near that of the standard.

FRAP Assay

In Figure 1c, the graph represents the antioxidant activity of* A. calamus *DMSO plant extract using the FRAP assay, in which the compound reduces ferric ions to ferrous ions. At the highest concentration of 50 µg/mL, the standard ascorbic acid shows a percentage of 90.89%, and the DMSO extract of *A. calamus* exhibits 87.48%. At a minimal concentration of 10 µg/mL, the *A. calamus *DMSO extract revealed 68.57%, and the standard ascorbic acid showed 72.55%. This shows that the drug efficacy was purely based on the dose-dependent concentration.

Anti-inflammatory

BSA Denaturation Assay

**Figure 2:**
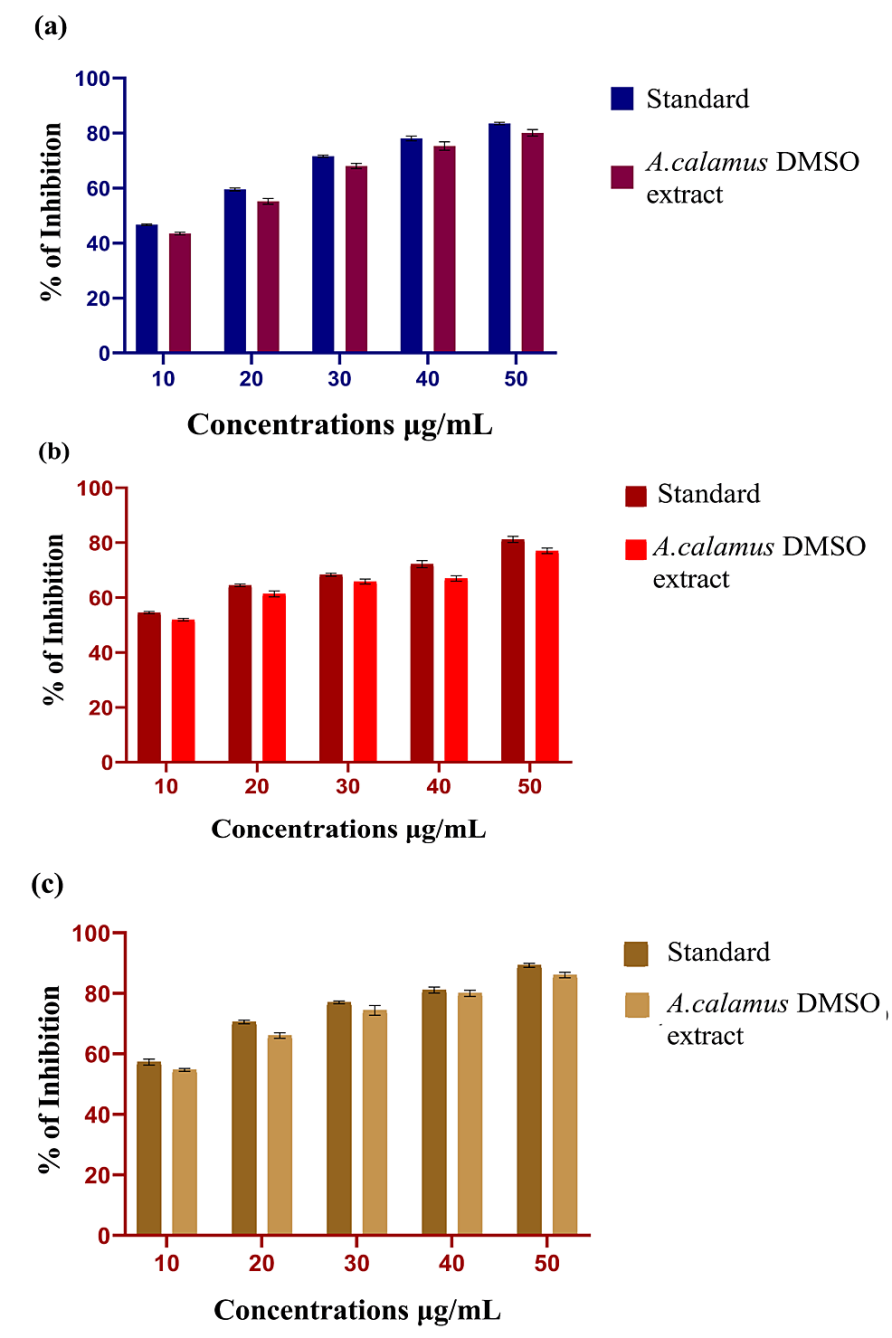
Anti-inflammatory activity of A. calamus DMSO extract (a) BSA denaturation assay, (b) EA denaturation assay, and (c) Membrane stabilization assay A. calamus: *Acorus calamus*; DMSO: Dimethyl sulfoxide; BSA: Bovine serum albumin; EA: Egg albumin The result values of the BSA, EA and membrane stabilization assays are provided in triplicate, and the error bars present in the bar graph indicate the standard error of mean (SEM). Welch's correction statistical test was performed to evaluate the significance. The value P≤0.05 was considered to be statistically significant.

The anti-inflammatory potential of *A. calamus* DMSO extract was assessed using the BSA denaturation assay. Various concentrations of the *A. calamus* DMSO extract were tested, and their inhibitory effects were compared to standard values. The findings revealed a 44% inhibition at a 10 μg/mL concentration and an 80% inhibition at 50 μg/mL. In comparison, the standard diclofenac sodium exhibited 47% inhibition at 10 μg/mL and 84% at 50 μg/mL. These results suggest that the *A. calamus* DMSO extract significantly inhibits the denaturation of BSA, demonstrating significant anti-inflammatory activity. Especially, the anti-inflammatory properties of the *A. calamus* DMSO extract were found to be comparable to those of the standard diclofenac sodium through all tested concentrations. In Figure 2a, the graph illustrates the anti-inflammatory activity of the DMSO extract of *A. calamus.*

EA Denaturation Assay

The anti-inflammatory activity of the *A. calamus* DMSO extract was assessed using the EA denaturation assay. The DMSO extract of *A. calamus* was evaluated at different concentrations and compared with standard (diclofenac sodium). The graph in Figure 2b displays the results of the EA denaturation assay, indicating a 52% inhibition rate at a concentration of 10 μg/mL. The standard diclofenac sodium exhibits a 58% inhibition rate, which is nearly identical to the standard percentage. At a volume of 50 μg/mL, the standard exhibits an inhibition percentage of 81%, while the *A. calamus *DMSO extract shows a similar inhibition percentage of 77%, which is close to the standard's inhibition percentage. The results indicate that the *A. calamus* DMSO extract shows substantial anti-inflammatory effects in the EA denaturation assay. In addition, the *A. calamus* DMSO extract exhibited anti-inflammatory characteristics that were equivalent to those of a standard, at all concentrations. Consequently, the *A. calamus* DMSO extract reveals promising anti-inflammatory efficacy in the EA assay.

Membrane Stabilization Assay

Figure 2c indicates the anti-inflammatory activity of the *A. calamus* DMSO extract, as determined by the membrane stabilization assay using HRBC (Human red blood cells). The DMSO extract of *A. calamus* was evaluated at various concentrations and compared to the standards. The results from this study suggested that the standard exhibited a 58% inhibition rate at a concentration of 10 μg/mL, while the extract showed a 55% inhibition rate. At the maximum concentration of 50 μg/mL, the standard diclofenac sodium exhibits an inhibition percentage of 89%, while the *A. calamus *DMSO extract shows a percentage inhibition of 86%. The results indicate that the *A. calamus* DMSO extract has a prominent anti-inflammatory effect in the membrane stabilization assay.

Antimicrobial activity

Agar Well Diffusion Method

**Figure 3:**
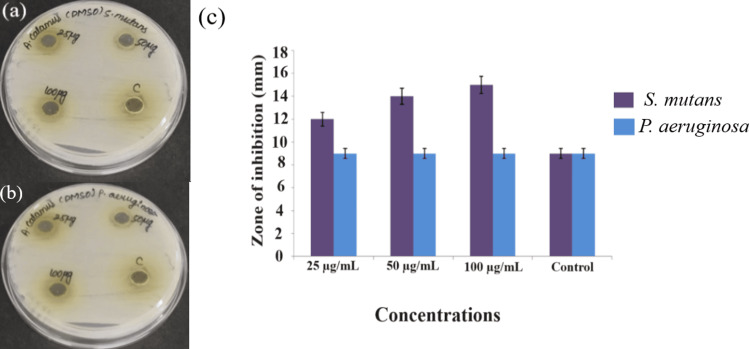
The image represents A. calamus DMSO extract Agar well diffusion method against the pathogens (a) S. mutans, (b) P. aeruginosa, and (c) graph illustrates the zone of inhibition of the bacterial strains A. calamus: *Acorus calamus*;* *DMSO: Dimethyl sulfoxide; S. mutans: *Streptococcus mutans*; P. aeruginosa: *Pseudomonas aeruginosa* The values for zone of inhibitionin in agar well diffusion method are provided in triplicate, and the error bars present in the graph indicate the standard error of mean (SEM). The Welch's correction statistical test was performed to evaluate the significance. P≤0.05 was statistically significant.

The antimicrobial efficacy of the *A. calamus* DMSO extract was assessed through the agar well diffusion method (Figures 3a, 3b), and the graph was depicted the zone of inhibition (Figure 3c). The DMSO extract obtained from *A. calamus* provided a zone of inhibition measuring 15 mm when 100 µg/mL of the extract was loaded and tested against *S. mutans* (Figure 3a). In comparison, the standard exhibited a zone of inhibition measuring 9 mm. The DMSO extract of *A. calamus *exhibited a 9-mm zone of inhibition when 100 µg/mL of the DMSO extract of *A. calamus* was tested against *P. aeruginosa *(Figure 3b). And the standard also showed a 9-mm zone of inhibition. The *P. aeruginosa *exhibited comparable levels of inhibition for both bacterial strains throughout three separate concentrations. Comparative to the commercial antibiotic Amoxyrite, the DMSO extract of *A. calamus* exhibited a greater zone of inhibition of 15 mm *S. mutans *at a concentration of 100 µg/mL.

Time-Kill Curve Assay

**Figure 4:**
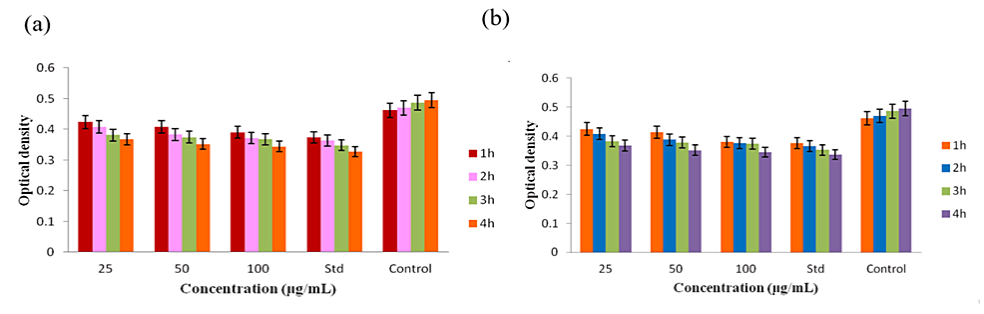
The graph indicates the Time kill curve assay of A. calamus DMSO extract against the bacterial strains (a) S. mutans and (b) P. aeruginosa A. calamus: *Acorus calamus*; DMSO: Dimethyl sulfoxide; S. mutans: *Streptococcus mutans*; P. aeruginosa: *Pseudomonas aeruginosa* The results are provided in triplicate. The error bars present in a graphical representation of the Time kill curve assay indicate the standard error of mean (SEM). The statistical test used was Welch's correction. A value of P≤0.05 was considered to be statistically significant

The time-kill curve assay showed that the DMSO extract of *A. calamus* exhibited antibacterial effects that were dependent on the concentration, as compared to the control group. At all concentrations of *A. calamus *DMSO extract (25 µg/mL, 50 µg/mL, and 100 µg/mL), there was a significant decrease in the amount of *S. mutans* and *P. aeruginosa* (Figure 4a). Significantly, when the concentration reached its peak at 100 µg/mL, a substantial decrease in *S. mutans* colonies was noted, indicating swift bactericidal efficacy. Figure 4b demonstrates that, unlike *S. mutans*, the strain *P. aeruginosa* exhibited a strong antibacterial effect. *A. calamus *DMSO extract caused a significant reduction in *P. aeruginosa* counts at all concentrations when compared to the control group. Especially at the highest concentration, there was a notable diminution. The results highlight the antibacterial properties of *A. calamus *DMSO extract against the tested bacterial strain, which vary depending on the concentration.

## Discussion

The results obtained from the assays evaluating the antioxidant, anti-inflammatory, and antibacterial properties of the *A. calamus *DMSO extract offer useful information about its possible therapeutic applications. The antioxidant capacity of the* A. calamus *DMSO extract was assessed using DPPH, H_2_O_2_, and FRAP assays in comparison to the effectiveness of the standard ascorbic acid. The* A. calamus* DMSO extract significantly blocked DPPH radicals in the DPPH assay, though it had slightly less activity than the standard at the highest concentration. This suggests strong antioxidant effects, which can be linked to the existence of bioactive components in the extract [17]. The antioxidant activity had been further verified by the H_2_O_2_ assay, which demonstrated a substantial decrease in both the extract and standard. The extract's performance was notably similar to the standard, indicating its potential as a natural antioxidant [18]. Both the extract and the standard showed dose-dependent antioxidant activity, as indicated by the FRAP assay, which evaluates the reduction of ferric ions. While the extract exhibited a somewhat lower reduction percentage compared to the standard, the general pattern indicates that the *A. calamus* DMSO extract has significant ferric ion-reducing activity [19]. The results highlight the potential of *A. calamus* DMSO extract as a powerful antioxidant, perhaps due to the presence of phenolic components and other antioxidants [20].

The anti-inflammatory activities of the *A. calamus *DMSO extract were measured by conducting BSA denaturation, EA denaturation, and membrane stabilization experiments while comparing its effects to the standard diclofenac sodium [21]. The BSA assay demonstrated a notable decrease in protein denaturation by the extract, similar to the standard, suggesting strong anti-inflammatory effects. The findings were further validated by the EA assay, which consistently showed a reduction of EA denaturation [22]. This suggests that the extract can regulate inflammatory reactions. The *A. calamus* DMSO extract showed notable anti-inflammatory properties in the membrane stabilization assay employing HRBC [23]. The inhibition rates observed at various concentrations were similar to those of the standard, indicating the extract's potential to prevent the instability of membranes that are linked to inflammation. These findings are consistent with the conventional applications of *A. calamus* in traditional medicine as an anti-inflammatory medication [24].

The agar-well diffusion method used in this study was used to initially evaluate the antibacterial effectiveness of the *A. calamus *DMSO extract. This method enables the qualitative assessment of the extract's capacity to inhibit the proliferation of particular bacterial strains [25]. The significant areas of inhibition identified, especially against *S. mutans*, emphasize the extract's strong antimicrobial effectiveness. The remarkable aspect to highlight is the immense efficacy of the *A. calamus* DMSO extract in comparison to the usual antibiotic. This indicates that the extract may include a powerful combination of bioactive chemicals that specifically target the tested bacterial strains [26].

The high inhibitory impact on *S. mutans*, a primary causative agent of dental caries, holds significant implications for possible utilization in oral health. The presence of an increased zone of inhibition indicates that the extract may contain substances that may inhibit the growth of bacteria or interfere with crucial microbial activities [27]. The time-kill curve assay offers a good viewpoint on the antimicrobial activity of the *A. calamus* DMSO extract, providing insights into whether the extract is bactericidal or bacteriostatic over time. The extract's prolonged antimicrobial action is revealed by the decrease in viable counts of bacterial colonies, which is concentration dependent. This is demonstrated by the substantial reduction in bacterial numbers observed at higher doses, which indicates a response that is dependent on the dosage. The extract's efficacy against both *S. mutans* and *P. aeruginosa* indicates a wide-ranging antibacterial capability. The concentration-dependent response indicates that the extract's antibacterial activity is both significant and adjustable according to the dosage [28]. The notable attribute of this substance is its ability to effectively kill both gram-positive (*S. mutans*) and gram-negative (*P. aeruginosa*) bacteria. This is particularly remarkable because gram-negative bacteria are generally difficult to eliminate due to their complex cell wall construction [29].

Limitations

This research explores the therapeutic potential of *A. calamus* DMSO extract, but it also identifies certain limitations. The study does not identify specific bioactive compounds, which are necessary for understanding their mechanisms. Additionally, the study heavily relies on in vitro experiments, which need to be validated in vivo settings to thoroughly assess safety and efficacy. The narrow emphasis on *A. calamus *restricts broader comparisons, and the observed responses that vary with concentration necessitate a more detailed investigation of dose-response relationships. Moreover, the limited evaluation of antimicrobial efficacy against only two bacterial strains (*S. mutans* and *P. aeruginosa*) hinders the ability to apply the findings to a wider range of situations. Although it provides fundamental insights, it is crucial to address these limitations in future research to enhance our comprehensive understanding of the extract's therapeutic uses.

## Conclusions

The experiments' results jointly emphasize the varied pharmacological actions of the *A. calamus* DMSO extract. The extract exhibited robust antioxidant, anti-inflammatory, and antibacterial characteristics, demonstrating effectiveness that was equal to or even exceeded that of conventional chemicals in certain cases. These findings corroborate the conventional applications of *A. calamus* in alternative medicine and establish a scientific groundwork for its therapeutic capacity. Additional research is necessary to isolate and identify the specific bioactive chemicals responsible for these effects. In addition, in vivo trials should be conducted to validate and build upon these promising results. The DMSO extract of *A. calamus* shows potential as a natural source of bioactive chemicals that could be used in the pharmaceutical and nutraceutical industries.
